# What are medical students taught about persistent physical symptoms? A scoping review of the literature

**DOI:** 10.1186/s12909-024-05610-z

**Published:** 2024-06-04

**Authors:** Catie Nagel, Chloe Queenan, Chris Burton

**Affiliations:** Primary Care Research Group, Division of Population Health, School of Medicine, University of Sheffield, Regent Court, 30 Regent Street, S1 4DA, Sheffield, UK

**Keywords:** Scoping review, Narrative synthesis, Undergraduate, Persistent physical symptoms, Medically unexplained symptoms, Chronic pain

## Abstract

**Background:**

Persistent Physical Symptoms (PPS) include symptoms such as chronic pain, and syndromes such as chronic fatigue. They are common, but are often inadequately managed, causing distress and higher costs for health care systems. A lack of teaching about PPS has been recognised as a contributing factor to poor management.

**Methods:**

The authors conducted a scoping review of the literature, including all studies published before 31 March 2023. Systematic methods were used to determine what teaching on PPS was taking place for medical undergraduates. Studies were restricted to publications in English and needed to include undergraduate medical students. Teaching about cancer pain was excluded. After descriptive data was extracted, a narrative synthesis was undertaken to analyse qualitative findings.

**Results:**

A total of 1116 studies were found, after exclusion, from 3 databases. A further 28 studies were found by searching the grey literature and by citation analysis. After screening for relevance, a total of 57 studies were included in the review. The most commonly taught condition was chronic non-cancer pain, but overall, there was a widespread lack of teaching and learning on PPS. Several factors contributed to this lack including: educators and learners viewing the topic as awkward, learners feeling that there was no science behind the symptoms, and the topic being overlooked in the taught curriculum. The gap between the taught curriculum and learners’ experiences in practice was addressed through informal sources and this risked stigmatising attitudes towards sufferers of PPS.

**Conclusion:**

Faculties need to find ways to integrate more teaching on PPS and address the barriers outlined above. Teaching on chronic non-cancer pain, which is built on a science of symptoms, can be used as an exemplar for teaching on PPS more widely. Any future teaching interventions should be robustly evaluated to ensure improvements for learners and patients.

## Background

Persistent Physical Symptoms (PPS) are symptoms which are disproportionate to currently recognised pathology and are common in all fields of medicine. The term encompasses single symptoms such as pain, dizziness or fatigue, and established syndromes including fibromyalgia and irritable bowel syndrome. It is increasingly understood that PPS arise from complex interactions between the brain and body [1, 2]. While historically terms such as “medically unexplained symptoms” have been in common use, most symptoms can actually be explained [3] and PPS is a more acceptable term to patients [4].

PPS are common and present to nearly every medical specialty. They represent the primary reason for presentation in around 45% of general practice consultations and between 30 and 70% of presentations to neurology, gynaecology, and rheumatology outpatient clinics [5]. People with PPS suffer unduly in a medical system that is predisposed to ‘body part medicine,’ [6] resulting in what Balint referred to as the “collusion of anonymity.” [7] In other words, patients who pass from specialist to specialist, without any doctor taking full responsibility for holistic care. Patients with PPS consult more frequently [8] and tend to have a higher rate of referral to secondary care [9]. This is costly, both in financial terms and in terms of the emotional work for patients and clinicians [10, 11]. Patients with PPS often have a poor experience of the health system and can be left feeling marginalised and even stigmatised [12]. 

Doctors find it difficult to consult and manage patients with persistent physical symptoms [8]. The absence of a common language of explanation to reconcile patients’ lived experience with doctors’ biomedical models, is particularly problematic [13]. It is plausible that difficulties may arise, or be perpetuated by, issues in each of the three domains of learning: cognitive (knowledge), psychomotor (skills) and affective (attitudes) [14]. 

The shifting perspectives, particularly around “medically unexplained symptoms” may account for historical uncertainty, however recent adoption of more consistent language and underlying models of symptoms mean that a common curriculum should be possible [15]. It is the authors’ experience that little teaching and learning at the undergraduate level has previously taken place on this topic. We wanted to find out if this was still the case, by reviewing the current medical education literature.

## Methods

We carried out a scoping review with narrative synthesis following the approach of Arksey & O’Malley [16]. The PRISMA-ScR guidelines were used to structure reporting [17]. 

### Research questions

The aim of the review was to explore the published literature regarding undergraduate medical teaching and learning on persistent physical symptoms. The specific research questions were:

What teaching and learning on persistent physical symptoms has been described for medical undergraduates?

What teaching methods have been used and how have these been evaluated?

### Search strategy

We used a Population, Concept, and Context (PCC) framework to structure a systematic search. The population was undergraduate medical students, the concept was persistent physical symptoms, and the context was teaching and learning. A variety of synonyms were used in order to be inclusive, given the constant evolution of terms for persistent physical symptoms. We used adjacency searching and truncation methods in order to broaden the search as widely as possible and to account for different spellings of words or use of phrases across the international context. Search concepts were then combined using Boolean operators. No date range was used, so all studies before 31 March 2023 were included. Inclusion criteria were: studies relating to the teaching and learning of Persistent Physical Symptoms; medical students included in the population; available in the English language. Exclusion criteria were: studies about cancer or terminal pain without the inclusion of other forms of chronic or persistent pain; population not including medical students; letters to the editor, and papers which were not available in the English language. See Table 1 for the full search strategy.

**Table 1:** Search terms used

Population	Condition	Context
Medical student	(Persistent adj2 symptoms)	Education
Medical students	(Functional adj2 symptoms)	Teach*
Student doctor	(Functional adj2 syndrome)	Learn*
Intern*	Medically Unexplained Physical Symptoms	Curriculum
Physician	Medically Unexplained Symptoms	Curricula
Medic	Non-organic	
Undergraduate medic*	Somatisation	
Doctor in training	Somatization	
	Fibromyalgia	
	Chronic pain	
	Chronic primary pain	
	Functional Pain	
	Chronic fatigue	
	Chronic fatigue syndrome	
	Chronic widespread pain	
	Myalgic Encephalopathy	
	Myalgic Enchphalomyelitis	
	(Functional adj2 gut)	
	Disorder gut brain interaction	
	Irritable bowel syndrome	
	Persistent somatic symptoms	
	Bodily distress disorder	
	Bodily distress syndrome	
	Functional somatic disorder	
	Somatic symptom disorder	

## Sources of evidence

Two authors searched for published literature in MEDLINE, PsycINFO, and Web of Science. Additionally, we searched Google and Google Scholar in order to include any grey literature or sources that had not been picked up by the previous search method. We employed citation analysis, by following backward citations from included papers and analysed the citations of any existing literature reviews.

### Study selection

We used a two-stage screening process to identify eligible papers: first at title and abstract level and then at full text. This method was undertaken separately by two reviewers.

Literature reviews were excluded to avoid duplicated representation of primary data, but citations in these reviews were analysed to ensure consistent inclusion of studies and to check for any additional sources.

### Charting the data: summary and synthesis

Summary findings for each full text article were charted to determine the most relevant items for extraction. This was an iterative process given the high degree of heterogeneity between the studies. Charting was conducted by two reviewers independently. Discrepancies in charting and data extraction were discussed in review meetings and a consensus was reached regarding which data to include for analysis.

Reviewers extracted descriptive data including: country of origin, whether the study was experimental or observational, the characteristics of the study participants, and whether any teaching intervention was evaluated. Other study characteristics were noted, such as the symptom or syndrome represented, as well as the type of study or intervention.

The expectation was that there would be a lack of teaching and learning on the subject of persistent physical symptoms. For this reason, the scoping review aimed to capture the greatest breadth of studies, rather than exclude studies based on quality criteria. If a teaching intervention was used, we did look at whether this was evaluated using a validated tool.

Following the extraction of descriptive data, a narrative synthesis was undertaken to identify other key findings. An inductive, iterative approach was taken in order to identify themes relating back to the research question. Manual coding was undertaken by two authors independently, followed by a discussion with all authors to arrive at an interpretation of the findings.

## Results

### Search strategy, study selection, and data extraction

**Fig. 1:**
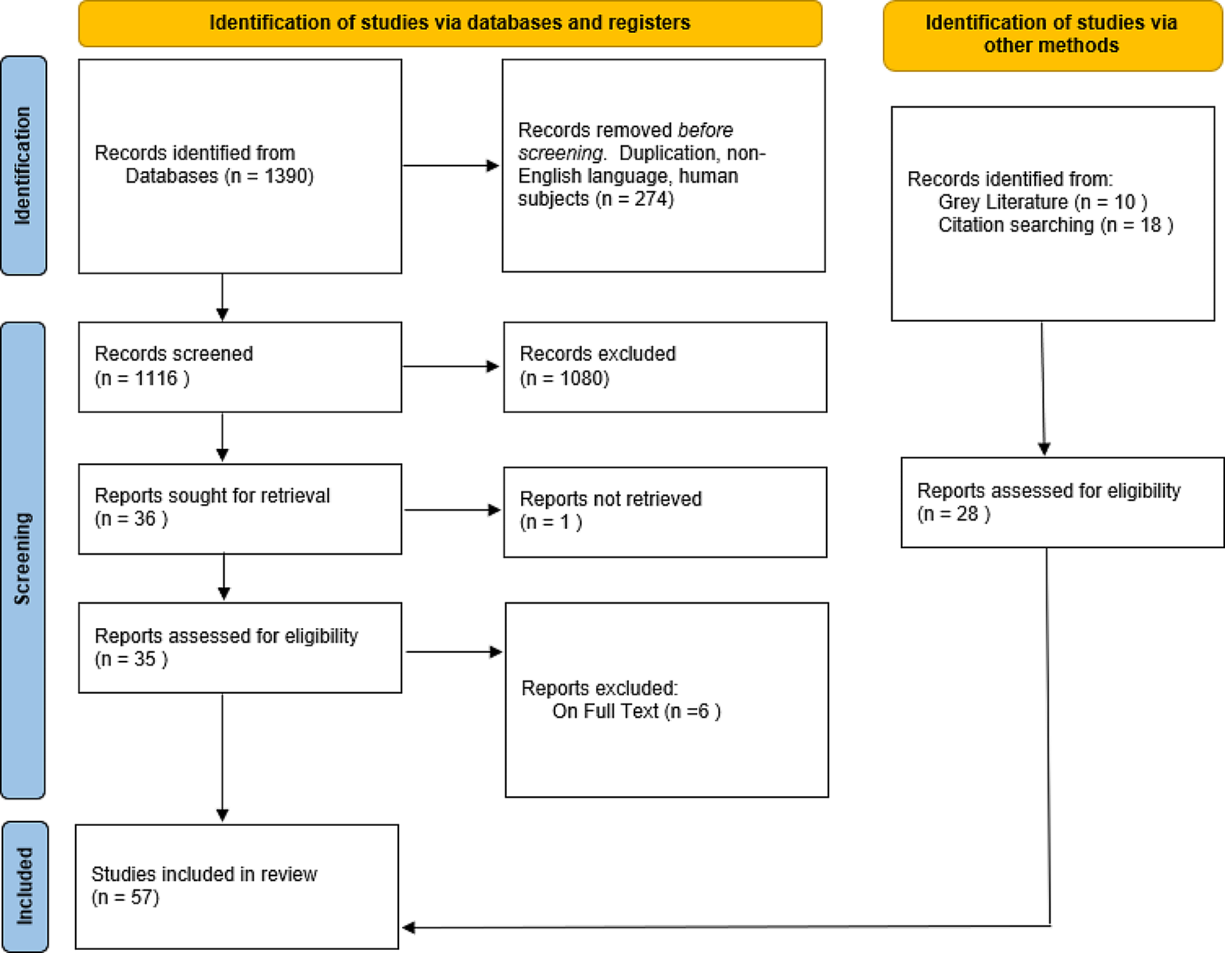
PRISMA Diagram Adapted from Page MJ, et al. [17]

Searches identified 1390 unique titles. Studies were limited to English language and human participants, leading to 274 being excluded. First stage screening excluded a further 1080 studies. It was not possible to retrieve one study and six were excluded on full text. Ten further records were identified through a grey literature search using Google and Google Scholar and 18 were found through citation searching, three of which were from a previous literature review [15]. This resulted in 57 publications for inclusion in the review. See the PRISMA flow diagram in Fig. 1 for a summary of these findings.

### Descriptive analysis

#### Study types

The studies included for review were highly heterogeneous in their nature. 15/57 (26%) studies employed a teaching intervention, with the remaining either being observational or qualitative. 8/57 (14%) studies described or evaluated the teaching curriculum, 13/57 (22%), included an assessment of the current level of learner knowledge. 9/57 (16%) used qualitative methods with learners and 6/57 (11%) with medical educators. One literature review on assessing knowledge, perceptions and attitudes to pain was found [15]. The citations of this review were checked and the three new sources [18–20] were included for review. Sources within this literature review that did not meet the eligibility criteria were excluded. The findings of the review itself were noted for congruity, but not formally analysed.

#### Study characteristics

23/57(40%) of studies took place in USA and 13/57 (23%) in the UK. Six studies took place in Scandinavia and four in Canada, four in Australia and one in New Zealand, India, and Nigeria respectively. Some studies were based in more than one country e.g. Australia and New Zealand [21]. Publication dates ranged from 1992 to 2022. See Table 2 for a summary of the descriptive data.

**Table 2:** Summary of included studies

Author	Year	Country	Topic	Study Type
Wilson, J [27]	1992	USA	Chronic Pain	Teaching Intervention
Turner, W [54]	2002	USA	Chronic Pain	Teaching Intervention
Watt-Watson, J [20]	2004	Canada	Chronic Pain	Teaching Intervention
Niemi-Murola, L [55]	2006	Finland	Chronic Pain	Teaching Intervention
Friedberg, S [22]	2008	USA	Medically Unexplained Symptoms	Teaching Intervention
Stevens DL, K [47]	2009	USA	Chronic Pain	Teaching Intervention
Murinson, BB [19]	2011	USA	Chronic Pain	Teaching Intervention
Morley-Forster, P [42]	2013	USA and Canada	Chronic Pain	Teaching Intervention
Weiner, D [39]	2014	USA	Chronic Pain	Teaching Intervention
Saypol, B [56]	2015	USA	Disorder of Gut Brain Interaction	Teaching Intervention
Bradshaw, YS [57]	2017	USA	Chronic Pain	Teaching Intervention
Bradner, M [48]	2019	USA	Chronic Pain	Teaching Intervention
Vargovich, A [34]	2019	USA	Chronic Pain	Teaching Intervention
Leeds, F [58]	2020	USA	Fibromyalgia	Teaching Intervention
Gadde, U [59]	2020	USA	Chronic Pain	Teaching Intervention
Campbell, W [60]	1992	UK (Ulster)	Chronic Pain	Survey
Chibnall, J [61]	1997	USA	Chronic Pain	Survey
Jason, LA [32]	2002	USA	Chronic Fatigue Syndrome	Survey
Pöyhiä, R [40]	2005	Finland	Chronic Pain	Survey
Niemi-Murola, L [62]	2007	Finland	Chronic Pain	Survey
Ali, N [45]	2009	UK	Chronic Pain	Survey
Howman, M [33]	2012	UK	Medically Unexplained Symptoms	Survey
Morris, H [23]	2012	UK	Chronic Pain	Survey
Briggs, AM [18]	2013	Australia	Chronic Pain	Survey
Murinson, BB [41]	2013	USA	Chronic Pain	Survey
Tauben, D [46]	2013	USA	Chronic Pain	Survey
Amber, K [63]	2014	USA (Miami)	Fibromyalgia	Survey
Amber, K [64]	2014	USA	Fibromyalgia	Survey
Adillón, C [65]	2015	Spain	Chronic Pain	Survey
Argyra, E [66]	2015	Greece	Chronic Pain	Survey
Briggs, E [67]	2015	Europe	Chronic Pain	Survey
Hollingshead, N [68]	2015	USA	Chronic Pain	Survey
Rankin, L [69]	2018	Sweden and Australia	Chronic Pain	Survey
Shipton, E [21]	2018	Australia & NZ	Chronic Pain	Survey
Baessler, F [49]	2019	Germany	Other	Survey
Cristóvão, I [70]	2019	Portugal	Chronic Pain	Survey
Gustafsson, S [71]	2019	Sweden	Chronic Pain	Survey
Lechowicz, K [72]	2019	Poland	Chronic Pain	Survey
Rankin, L [24]	2019	Australia and Sweden	Chronic Pain	Survey
Storrar, A [73]	2019	UK	Functional Neurological Disorders	Survey
Wojtowicz, A [43]	2020	USA	Disorder of Gut Brain Interaction	Survey
Muirhead, N [74]	2021	UK	Chronic Fatigue Syndrome	Survey
Emorinken, A [36]	2022	Nigeria	Fibromyalgia	Survey
Simons, J [75]	2022	UK	Disorder of Gut Brain Interaction	Survey
Lempp, H [51]	2010	UK	Chronic Pain	Qualitative
Corrigan, C [26]	2011	USA	Chronic Pain	Qualitative
Shattock, L [30]	2013	UK	Medically Unexplained Symptoms	Qualitative
Lambson, R [76]	2015	UK	Chronic Fatigue Syndrome	Qualitative
Stenhoff, S [31]	2015	UK	Chronic Fatigue Syndrome	Qualitative
Comer, L [37]	2017	Canada	Chronic Pain	Qualitative
Dwyer, C [44]	2017	Ireland	Chronic Pain	Qualitative
Silverwood, V [29]	2017	England	Fibromyalgia	Qualitative
Joyce, E [25]	2018	UK	Medically Unexplained Symptoms	Qualitative
Raber, I [77]	2018	USA	Chronic Pain	Qualitative
Rice, K [78]	2018	Canada	Chronic Pain	Qualitative
Vasanthy, B [28]	2018	India	Fibromyalgia	Qualitative
Sallay, V [79]	2022	Hungary	Medically Unexplained Symptoms	Qualitative

#### Teaching and learning methods

A wide range of teaching and learning methods were discussed in the literature. These are fully described in Table 3, but included lectures, workshops, reflective practice, and forum theatre.

**Table 3:** Summary of teaching interventions

Author	Year	Teaching Method	Evaluation Method	Outcome
Wilson, J	1992	Interdisciplinary course focused on chronic complex pain	Survey assessment of knowledge and attitudes towards chronic pain immediately following the course and 5 months later	Increased acceptance that the symptom was ‘real.’ Learners felt that treating pain was difficult, challenging, or unpleasant before and after the course.
Friedberg, F	2008	Interactive seminar using video, lecture, and discussion.	Measurement of attitudes before and after the teaching using Chronic Fatigue Syndrome Attitudes Test (CFSAT). Paired Statistical analysis of pre and post session scores.	Significantly more favourable attitudes about CFS following the seminar. Particularly supporting more research funding, employers providing flexible hours, and viewing it as not a primarily psychological disorder.
Stevens, D	2009	Experiential curriculum in second year focused on pain assessment and management. Teaching included chronic pain.	OSCE assessment on acute (shoulder) pain and terminal (cancer) pain.	Intervention cohort had improved scores in the terminal pain case.
Lempp, H	2010	Student Selected Component using art and placements to teach about chronic pain	Reflective diaries and co-production of a piece of visual art with a patient with chronic pain.	All students passed the module. Learners understood the importance of listening to patients.
Corrigan, C	2011	Reflective practice on chronic pain following clinical placement in a rural setting	Qualitative analysis of reflective log entries	Learners found chronic pain challenging. They were concerned about opiate use.
Morley-Forster, P	2013	A review of 3 undergraduate teaching initiatives on chronic pain. Variety of methods including workshops, e-learning, and lectures.	A case study approach was taken to the review. Evaluation not documented.	Experiential, multidisciplinary teaching and learning recommended.
Tauben, D	2013	4-year integrated pain curriculum	Knowledge based questionnaire survey before the change to curriculum. No post intervention analysis.	An increase of core pain related teaching from 6 h to 25
Weiner, D	2014	E-learning module on chronic low back pain in older adults	OSCE assessment with subsequent statistical analysis comparing learners who had done the module with those who had been subject to usual teaching.	Statistically significant improvement in the raw pass mark from 61–96%
Argyra, E	2015	Course on chronic pain	Knowledge based survey for participants who attended a chronic pain course. Compared with those who hadn’t	Improved knowledge and awareness of pain clinics in learners who attended the course
Saypol, B	2015	Interactive Theatre to improve communication skills in patients with irritable bowel syndrome.	Survey of learner feedback including satisfaction ratings.	Improved cultural competence and active listening skills. More appreciation of the importance of empathy and respect for others.
Dwyer, C	2017	Concept mapping exercise using interactive management (IM) with learners to determine factors influencing a biopsychosocial approach to assessing chronic pain.	n/a Interactive Management is a systems thinking and action mapping strategy.	7 categories identified: GP attitudes, cost, GP knowledge, time, patient-doctor relationships, biomedical factors, patient perceptions. GP attitude was deemed most influential.
Bradner, M	2019	Interactive teaching session with a simulated patient. Case based discussion.	Learner reflections. These were optional and not formally analysed using qualitative methods.	Learners were more empathic towards the simulated patient and viewed her as a whole person. The simulated patient was negatively affected by the stigmatised responses initially voiced by learners.
Vargovich, A	2019	Didactic lecture & workshop using standardised simulated patients	Pre and post workshop survey	Improvement in knowledge, attitudes, and confidence in managing chronic non-malignant pain
Gadde, M	2020	Didactic lecture and case-based learning, followed by experience of complementary therapies.	Pre and post session survey of self-reported knowledge. Analysed using paired t-test.	Improved awareness of complementary therapies for treating chronic pain.
Leeds, F	2020	13-minute patient experience video	Knowledge and attitudinal questionnaires before and after the video.	Learners preferred the video to a lecture. Improved knowledge and attitudes, particularly in understanding of the mechanisms and management of fibromyalgia

#### Evaluation of teaching studies

Four studies used validated tools to assess learner attitudes towards patients with PPS, but only one used such a tool to evaluate a teaching intervention [22]. Morris, Rankin, and Briggs used the HC-PAIRS attitudinal questionnaire to assess learner attitudes towards patients with chronic low back pain [18, 23, 24]. Whereas Friedberg et al. [22] used the Chronic Fatigue Syndrome Attitudes Test (CFSAT) and paired t-test to analyse learner attitudes before and after a teaching intervention. The remaining educational interventions either did not use a validated tool for evaluation or were not formally evaluated. See Table 3 for more details.

#### Thematic synthesis

All studies identified a lack of teaching about persistent physical symptoms (PPS) at undergraduate level. The narrative synthesis identified four themes: An awkward problem, an absence of science, being easily overlooked, and a hidden curriculum.

#### An awkward problem

PPS was consistently viewed as an awkward problem. Medical educators and learners found it difficult to understand, particularly when referring to the symptoms as ‘unexplained.’ Some educators described PPS as too complex or too confusing, even ‘dangerous’ to introduce at an undergraduate level and stated the need to focus on the easily ‘explainable.’ [25] Chronic non-cancer pain was the dominant condition represented in the literature, but despite theoretical concepts of chronic pain being more established, learners found the subject challenging, even ‘unpleasant.’ [26, 27].

#### The absence of science

Four studies highlighted that learners infer patients with PPS have ‘no science’ behind their symptoms. In the study by Vasanthy [28], clinical role models in Kerala were found to have a ‘nihilistic’ attitude towards people with fibromyalgia and regarded the condition as benign and unimportant. This finding was echoed by UK studies [29–31] where the impact of a lack of teaching and negative role modelling was evident:

*“You can’t really train someone for it because there is no science behind it”* [30].

One final year medical student stated that fibromyalgia was *“not a medical issue”* intimating that it had no place in the taught curriculum [29]. Learners understood the need to be supportive and for good communication, but only as a way of achieving relational congruence, not epistemic congruence [8]. Terminology may be important and in one study learners’ attitudes towards PPS varied depending on the diagnostic label [32]. As an example, learners thought that people with myalgic encephalopathy were less likely to recover than those with chronic fatigue syndrome [32]. 

#### Easily overlooked

Even without the overt attitudinal barriers described in some studies, PPS as a topic is overlooked in undergraduate medical education. The most common barrier was an already overloaded teaching curriculum [25, 33]. PPS was not deemed a priority area by educational leaders and [33] even when they recognised its importance, they cited a lack of ownership of the topic and a lack of coordination between teaching specialties as a barrier to implementing teaching. This was in contrast to chronic non-cancer pain teaching which usually did have clear ownership by pain specialists and established interdisciplinary relationships [34]. The experience of learners in the clinical setting was that they were shielded from patients with PPS or directed towards patients with other more easily defined clinical problems [28]. 

#### Stigma and the hidden curriculum

Given the vacuum of formal teaching, learners were taking on stigmatised messages about sufferers of PPS, frequently from role models in the clinical placement setting. Stenhoff and colleagues described a cycle of negativity created by the lack of teaching on the subject of chronic fatigue, which resulted in negative behaviour by clinical role models, in turn perpetuating negative attitudes in the next generation of learners [31]. Whilst learners recognised the problematic nature of the attitudes towards people with PPS, they lacked the tools to challenge negative stereotypes [29, 30, 35]. Learners experienced a mismatch between formal teaching on the topic and their experience on placement, where these conditions were frequently encountered. They addressed the gap by seeking information about PPS from informal sources, such as their own or their families’ experiences or from the internet [36]. This lack of explicit teaching and the influence of informal sources has been termed by some authors as the ‘hidden curriculum’ [29–31, 36] and this has had a significant impact on learners’ attitudes towards people suffering with PPS.

#### Suggestions for improvement: relationship to domains of learning

The findings of the narrative synthesis map onto Bloom’s revised three domains of learning [14]. 

#### Knowledge (cognitive)

A number of studies demonstrate success in teaching on the topic of chronic non-cancer pain. Teaching interventions tended to include a foundation of knowledge such as teaching on pain mechanisms, pharmacology, and pain management [37, 38]. Such theory-driven interventions led to improved scores on assessment [39]. Methods of teaching should be considered in the explicitly taught curriculum. Authors recommended an integrated approach [40, 41] and one which drew on the skills and knowledge from a variety of disciplines [37, 42]. Curriculum mapping was recommended by Howman et al. [33] in order to identify ways in which this integration could be implemented. The need for an holistic approach which emphasises the importance of empathy [41] and the biopsychosocial was also widely recognised [43–46]. Learners cited a lack of assessment as an indicator that PPS was either unimportant or uncommon [29, 33] and therefore any teaching intervention should include assessment in order to drive learning and engagement.

#### Skills (psychomotor)

Learners valued the addition of skills-based teaching and engaged best with teaching that was experiential [47] and included either patients with PPS or simulated patients [45, 48, 49]. In one study the focus of the teaching was on interactive, practical teaching for emotionally demanding consultations and the skills taught in such a programme could be transferable to the PPS context [49]. Approaches to help learners find a common language of explanation [13] will not only bridge the epistemic gap between clinicians and patients [8], but should give learners greater confidence and satisfaction in consultations where PPS are the focus.

#### Attitudes (affective) and the role of reflection

Reflection is a key transferable skill that graduates should acquire as part of their undergraduate training [50]. Both learners and educators voiced a great deal of anxiety regarding teaching and managing patients with PPS. Some authors utilised reflective logs and visual art as a way of teaching about chronic pain [51] and learners valued the deep insights provided by this method. Skills in reflection might help to ameliorate the negative emotions felt by learners, especially if combined with a taught framework that helps them understand concepts such as internal bias and cognitive dissonance [52].

## Discussion

### Summary of main findings

This review found that teaching on persistent physical symptoms in undergraduate medical education is inconsistent and incomplete. We identified four important themes: an awkward problem, the absence of science, easily overlooked, and the hidden curriculum. Mapping these to teaching and learning domains provides a coherent framework for undergraduate teaching of these common conditions. Where teaching does take place, this is more frequently on the topic of chronic non-cancer pain. A number of studies have demonstrated improved knowledge [39], skills [49], and attitudes [51] as a result of this teaching [34, 47], but high quality evaluation of such teaching and learning is lacking.

### Strengths and limitations

This scoping review has addressed a gap in the literature. By undertaking a search of three databases, the grey literature, and citation analysis, a wide range of sources were included for initial screening. Two researchers independently undertook the search strategy before comparing findings which has helped to ensure a robust and systematic approach. Narrative synthesis was undertaken by three researchers, one with expertise in the field of persistent physical symptoms.

The majority of the studies identified were from the USA and UK. Papers that were not accessible in English were excluded, which may explain this finding. Where teaching and learning evaluations had taken place, this was on a small scale usually within one institution. Only one study [22] used a validated tool to evaluate the efficacy of the teaching intervention.

### Implications for practice, policy, and research

There is a lack of teaching on PPS in undergraduate medical education. As a result, medical graduates are ill-equipped to recognise, consult for, and manage this group of conditions. Given the prevalence of PPS across medical specialties this is a priority area that needs to be addressed, whilst acknowledging the barriers that exist to implementation.

The solutions offered up in the literature include the need to consider whole-person care, in order to avoid fragmentation and the “collusion of anonymity” [7] described above. For this reason, teaching on PPS should be integrated into the core curriculum and draw on a variety of disciplines.

A better understanding of the science behind PPS [1, 2] is needed for both educators and learners. There is also a need to move learners beyond reductionist models of communication skills towards more theory-driven approaches of person-centredness, as identified by Bansal [53]. We need to convey to learners that skilled communication is not about platitudes, but can make a difference to recovery and addresses the current epistemic gap between clinicians and their patients [8, 13]. 

Future educational research should focus on the most effective methods to improve the knowledge base of both educators and students and how best to evaluate the success of future teaching interventions. Skills in person-centred communication and explanation [3] need to be taught, alongside those in reflection and challenging prejudice.

## Conclusion

We identified four important themes which underpin the challenges of teaching medical undergraduates about persistent physical symptoms. Educational faculties need to find ways to integrate teaching into current programmes and work around the existing barriers to successful implementation and evaluation of teaching about these common and limiting conditions. Examples of successful teaching on chronic non-cancer pain were found in the literature. These tended to articulate the science behind symptoms and often included experiential elements. Such examples should be used to inform an approach for teaching about other forms of PPS. Importantly, robust evaluation that accounts for the complexity of the taught environment is needed to ensure our teaching is making a difference, both for our learners and the patients they will go on to encounter.

## Data Availability

All data generated or analysed during this study are included in this published article and its supplementary information files.
